# Adaptation, production, and biotechnological potential of cold-adapted proteases from psychrophiles and psychrotrophs: recent overview

**DOI:** 10.1186/s43141-020-00053-7

**Published:** 2020-07-28

**Authors:** Junaid Furhan

**Affiliations:** Department of Microbiology, SKIMS Medical College-Hospital, Bemina, Srinagar, Jammu and Kashmir 190017 India

**Keywords:** Cold-adapted proteases, Enzymes, Psychrophiles, and Psychrotrophs

## Abstract

**Background:**

Proteases or peptidases are an imperative class of hydrolytic enzymes capable of hydrolyzing large proteins into smaller peptides. The cold-adapted proteases show higher catalytic capacity in low temperatures as well as stability in alkaline conditions and appear as strong contenders for various applications in special industries.

**Main body:**

In the past few decades, the interest in cold-adapted microorganisms producing cold-adapted proteases has increased at an exciting rate, and many of them have emerged as important biotechnological and industrial candidates. Industrial proteases are largely supplied from various types of microorganisms than plant or animal sources. Among diverse microbial sources, psychrophiles and psychrotrophs inhabiting permanently or partially cold environments have appeared as rich sources of cold-adapted proteases.

**Short conclusion:**

The present review focuses on recent sources of cold-adapted protease producers along with the molecular adaptation of psychrotrophs and psychrophiles. The recent knowledge on production, kinetic properties, purification, and substrate specificity of cold-adapted proteases has been summarized. Recent advances in cold-adapted protease gene cloning and structural studies are also described. Moreover, the prospective applications of cold-adapted proteases are discussed which can help in evaluating their industrial potential.

## Background

Most of the Earth’s biosphere is covered by cold blanket, and temperature at such cold ecosystems typically tends to be below 5 °C. These cold habitats include to a great extent, oceans that envelop 70% of the Earth’s surface, polar regions contained by the Arctic circle, high peaks of Alps and rocky mountains, Himalayan regions, and different layers of Earth’s atmosphere, and to some degree—refrigerator, deep-freezers, and other cold appliances [1, 2]. All these natural and man-made habitats collectively cover 85% of the Earth and colonize a large number of cold-adapted microorganisms particularly archaea, bacteria, fungi, viruses, and yeasts, broadly subdivided as psychrophiles and psychrotrophs/psychrotolerants [3]. These cold-adapted microorganisms have proven to be more economical and eco-friendlier when compared with microorganisms operating at normal or higher temperatures. In the past three decades, the broad biotechnological potential of cold-adapted microorganisms and their enzymes has been robustly documented. A wide variety of commercial and industrially important enzymes especially amylases, lipases, and proteases have been sourced from cold-adapted microorganisms [4]. The worldwide industrial market for enzymes has reached just about $5.5 billion in 2018 [5], and the worldwide market for food enzymes alone has grown to $1.8 billion during 2017 [6]. Proteases from microbial sources are vital industrial enzymes accounting for 60% of the overall sale of enzymes globally and are known to hold the prime share of the detergent enzyme market [7].

**Fig. 1:**
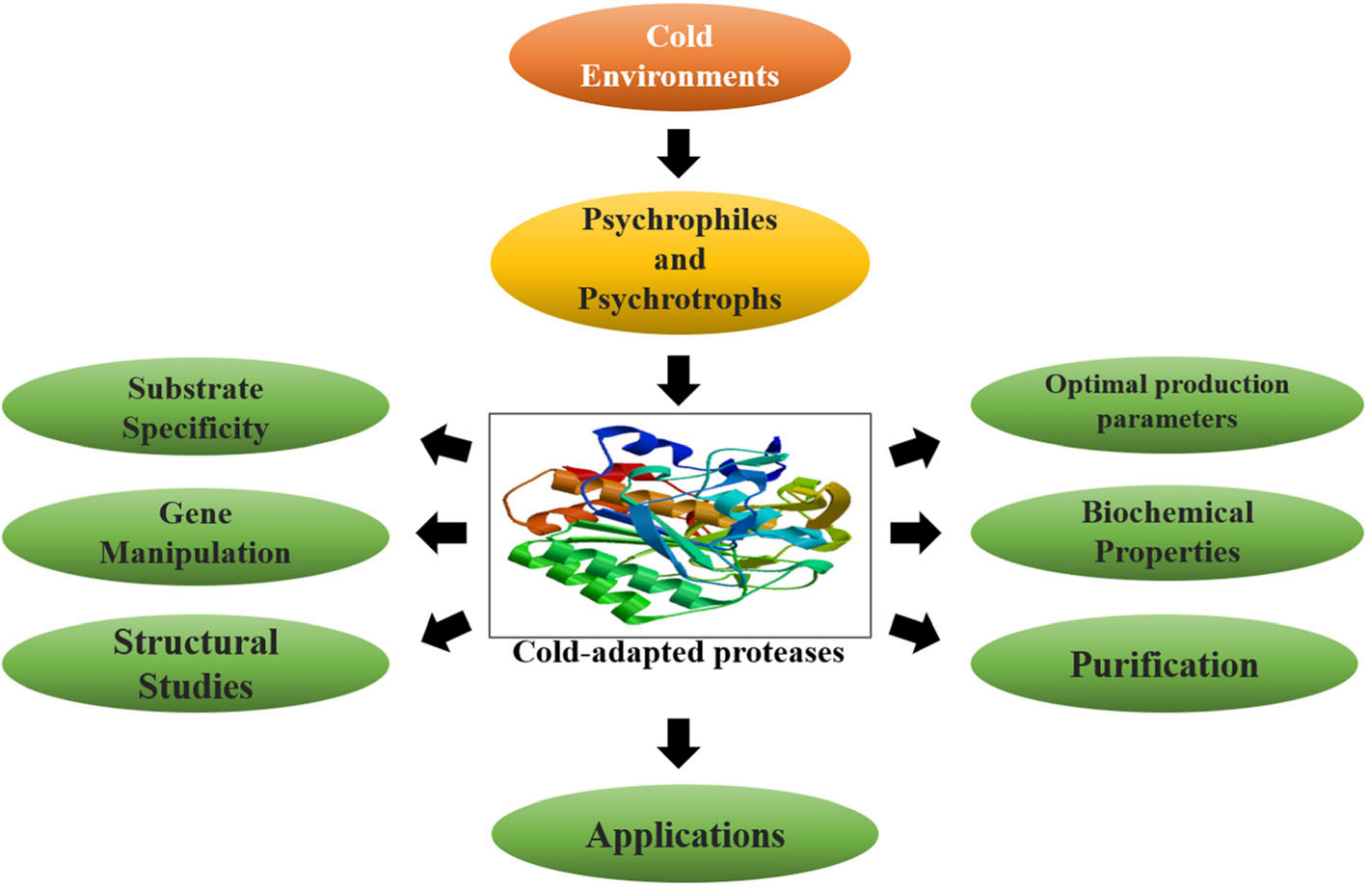
Flow diagram outlining the study criteria

Proteases represent imperative classes of hydrolytic enzymes that breakdown large proteins into smaller peptides and amino acids. Historically, proteases have been extensively used in detergent and food industries [8]. In recent times, proteases have shown biotechnological potential in a wide range of new industrial applications. Cold-adapted proteases represent vital a kind of enzymes; they have high catalytic efficiency at high temperatures and low thermostability in cold conditions which provides an advantage over the enzymes derived from mesophiles. Due to the advanced features of cold-adapted microorganisms and their cold-adapted enzymes, more attention has been paid towards the utilization of their potential for industrial applications during the last few years [9]. Researchers are continuously exploring various new aspects of cold-adapted proteases carrying novel properties to meet the rising demand of industrial sectors. Therefore, keeping the increasing literature in view, the present review summarizes the present status of recent resources, characteristics, and prospective applications of cold-adapted proteases from psychrophiles and psychrotrophs. Recent developments in gene cloning and structural studies of cold-adapted proteases which have proven to be one step forward in obtaining more robust proteases have also been discussed (Fig. 1).

## Main text

### Diverse sources of cold-adapted protease producing microorganisms

Since the first report on protease-producing psychrophilic *Escherichia freundii* of soil origin [10], not many protease-producing psychrophiles and psychrotrophs were detailed until the late 1990s. Since the beginning of the 21st century, work on cold-adapted proteases increased at an exciting rate, and they have been isolated and characterized by microbial diversities of cold-adapted bacteria, yeast, and fungi*.* The cold-adapted protease producers are not limited to low-temperature environments only but are spread nearly among all types of environments. In recent times, Antarctic cold habitats such as deep-sea sediment, Prydz Bay [11]; soil samples, King George Island [12–15]; seawater and krill [16]; marine water, Casey Station [17]; and penguin feathers [18] have been explored for isolation of cold-adapted protease-producing microorganisms. Other cold environments inhabiting cold-adapted protease-producing microorganisms are Japanese deep-sea water [19], yellow sea [20, 21], Ikka Fjord and surficial sediments in Greenland [22], deep-sea mud in Eastern Indian Ocean [23], and Bolu Mountain and Erzurum soil in Turkey [24, 25]. Cold-adapted protease producers have been isolated from soil samples of various Indian Himalayan regions such as Lahaul and Spiti [26], Kashmir apple garden [27], Thajiwas glacier [28], Gangotri glacier [29], and Wular Lake [30]. Other sources used for isolation of cold-adapted protease producers include an underground water sample of abandoned silver and lead mine [31] and cold food products and storage facilities [32]. Table 1 shows some recently isolated cold-adapted protease-producing microorganisms. Using a novel approach for enrichment, four different genera of cold-adapted protease-producing bacteria were isolated collectively from Norwegian marine and terrestrial samples [33]. Similarly, a swift and direct screening method was adapted, and fifteen cold-adapted protease-producing bacterial strains belonging to two different genera were isolated from the Arctic and Antarctic region [35]. Recently, the assortment of culturable psychrotrophic and psychrophilic bacteria from three sub-glacial Himalayan lakes was examined for cold-adapted enzymes and many cold-adapted protease-producing Bacilli belonging to different families were isolated [38].

**Table 1:** Cold-adapted microorganisms known to produce cold-adapted proteases (published from 2010 onwards)

Microorganisms producing cold-adapted proteases	Source of isolation	Reference
**Bacteria**		
*Acinetobacter* sp.	Himalayan cold soil	[26]
*Arsukibacterium ikkense*	Ikka Fjord, Greenland	[22]
*Arthrobacter* sp.	Arctic marine and terrestrial samples	[33]
*Bacillus* sp.	Antarctic soil	[12]
*Bacillus* sp.	Apharwat glacier	[34]
*Bacillus subtilis*	Wular Lake	[30]
*Chryseobacterium sp.*	Natural and artificially cold environments	[32]
*Flavobacterium*	Chinese Yellow Sea	[20]
*Flavobacterium* sp.	Arctic terrestrial soil	[33]
*Flavobacterium* sp.	Arctic and Antarctic region	[35]
*Flavobacterium* sp.	Antarctic water samples	[36]
*Halobacillus* sp.	Marine sea sediment	[37]
*Lysinibacillus fusiformis*	North-Western Himalaya	[38]
*Lysobacter* sp.	Antarctic Penguin feathers	[18]
*Mycoplana* sp.	Himalayan cold soil	[26]
*Planococcus* sp.	Deep-sea mud	[23]
*Planomicrobium* sp.	Deep-sea sediment	[11]
*Pseudomonas* sp.	Arctic terrestrial sample	[33]
*Pseudomonas* sp.	Himalayan cold soil	[26]
*Pseudomonas* sp.	Antarctic water	[36]
*Pseudomonas aeruginosa*	Yellow Sea	[21]
*Pseudomonas lundensis*	Sea sediment	[19]
*Pseudoalteromonas* sp.	Arctic marine sample	[33]
*Pseudoalteromonas* sp.	Arctic and Antarctic region	[35]
*Pseudoalteromonas* sp.	Antarctic seawater and krill	[16]
*Pseudoalteromonas arctica*	Antarctic soil	[13]
*Pseudoalteromonas haloplanktis*	Antarctic marine	[39]
*Pseudoxanthomonas* sp.	Himalayan cold soil	[26]
*Serratia* sp*.*	Himalayan cold soil	[26]
*Serratia marcescens*	Apple garden soil	[27]
*Stenotrophomonas* sp.	Himalayan cold soil	[26]
*Stenotrophomonas* sp.	Thajiwas glacier soil	[28]
*Stenotrophomonas maltophilia*	Gangotri glacier soil	[29]
**Fungi**		
*Geomyces pannorum*	Antarctica	[15]
*Penicillium nalgiovense*	Moldy sausage	[40]
**Yeast**		
*Cryptococcus victoriae*	Turkish soil	[25]
*Glaciozyma antarctica*	Antarctic marine water	[17]
*Goffeauzyma gilvescens*	Antarctic soil	[14]
*Naganishia globosa*	Antarctic soil	[14]
*Naganishia adeliensis*	Antarctic soil	[14]
*Naganishia albida*	Antarctic soil	[14]
*Sporobolomyces roseus*	Water of disused silver and lead mine	[31]
*Yamadazyma* spp.	Water, leaf, and grass from mountain	[24]

### Environmental adaptation and survival strategies of cold-adapted microorganisms

Psychrophiles and psychrotrophs have accumulated a multiplicity of approaches and mechanisms that help them in enduring and inhabiting environments that are exposed to permanent or partial cold temperatures [41]. The cold ecosystems expose microorganisms to harsh and unusual conditions were maintaining the structural integrity, and normal functioning of a cell is dependent upon the disposal of the fractional part of the water from the intracellular space [42]. There has not been any clear lower temperature limit for cold-adapted microorganisms; however, in a permafrost bacteria, reproduction has been reported at − 12 °C and metabolic function at − 20 °C [43]. Microbiologists have reported lichen *Umbilicaria aprina* from Antarctica that carries photosynthesis at − 17 °C [44], and yeast *Rhodotorula glutinis* that causes frozen food spoilage at − 18 °C [45]. To overcome the negative influence of cold temperatures on normal cellular functioning and interactions, several tactical approaches and mechanisms are maintained by cold-adapted microorganisms [46, 47]. They manage to curb ice crystal growth and ice recrystallization by producing small antifreeze or ice-binding (AFP) proteins which in turn lowers the freezing point by adsorbing to ice and arrest the process of freezing [48]. The ice-nucleating (IN) proteins can prevent the rapid cooling of water by inducing the crystallization of ice at temperatures close to the melting point [49]. Cold-shock proteins (CSPs) are small, single-stranded, and are encoded by the most important up-regulated gene and appear to be a further significant feature of cold-adapted microorganisms. They bind to nucleic acid and help in regulating a variety of cellular processes such as folding of proteins, transcription, translation, and fluidity of membranes [50, 51]. Another type of protein known as cold acclimation proteins (CAPs) is highly manufactured in cold-adapted microorganisms in response to low temperature for maintaining cell cycle and growth [51, 52]. Cold-adapted fungi and yeast have been reported to stockpile polyunsaturated fatty acids in high levels for maintaining membrane fluidity at low temperatures [53, 54]. Organic osmolytes or compatible solutes are accumulated by various cold-adapted microorganisms to prevent cell contraction and water loss by reinstating osmotic balance during freezing. Besides, they lower the freezing point and the colloidal glass transition temperature (Tg) and also stabilize proteins and membranes at lower temperatures [55]. Some cold-adapted bacteria produce multifunctional extracellular polymeric substances that play a significant role in restraining ice growth and ice-recrystallization. Moreover, they protect against osmotic stress and desiccation damage caused by freezing [56, 57]. Similarly, chaperons produced in some cold-adapted microorganisms help in promoting the folding and stability of proteins and also play a role in the deterioration of genetic material [58].

### Optimization of production parameters of cold-adapted microbial proteases

Traditionally, the production of cold-adapted proteases from cold-adapted microorganisms has been greatly influenced by nutritional, environmental, and physicochemical parameters [4, 59]. Owing to better management of environmental factors, submerged fermentation has been exploited more than solid-state fermentation for cold-adapted protease production. Approximately 40% of the production cost of industrial enzymes is accounted for by the usage of growth medium; therefore, it is important to identify and supply cost-effective media for the production of cold-adapted proteases [1]. There has not been any particular defined medium recognized for the optimal production of cold-adapted proteases as every cold-adapted microorganism has its own unique nutritional, environmental, and physicochemical requirements.

#### Source of growth media

Nutritional factors such as carbon and nitrogen sources are important components of media that stimulate microbial growth and protease production. Enhanced protease production by *Chryseobacterium* sp. was observed when starch (10 g/L) was used as the carbon source and urea (5 g/L) as a nitrogen source in the growth media [32]. Fructose in presence of skim milk was the best carbon source (2.4 fold increase), and tryptone in presence of skim milk (1.7-fold increase) was the best nitrogen source for protease production by *Pseudoalteromonas arctica* [13]. For psychrotrophic *Sporobolomyces roseus*, glucose was the best carbon source, whereas other sources like yeast extract, beef extract, and BSA showed a lower effect on protease production [31]. The highest cold-adapted protease production by psychrotolerant *Bacillus subtilis* [30], *Chryseobacterium* sp. [32], and *Stenotrophomonas maltophilia* [29] was observed in medium supplemented with casein as substrate source. Lactose and soya bean were the best carbon and nitrogen source respectively, whereas TSB was found to be the best medium for protease production by psychrotolerant *Stenotrophomonas* sp. [28]. For *Bacillus* sp., glucose was found to be the best carbon source, whereas skimmed milk was found to be the best substrate for optimal protease production [34].

#### Incubation time

Incubation time plays a vital role in affecting the protease production by various cold-adapted microorganisms, and they can be either growth-dependent or growth-independent. The cold-adapted protease production by *Chryseobacterium* sp. was observed to be growth-dependent, and the highest production was achieved after 6 days of incubation [32]. The *Cryptococcus victoriae* produced maximum cold-adapted protease for immobilized cells (13.4 U/ml) and free cells (12.1 U/ml) after 72 and 96 h of incubation respectively [25]. The extracellular protease production by *Planococcus* sp. started at an early stage of the stationary phase, increased progressively, and reached the maximum at the late stationary phase (48–56 h) [60]. Kuddus and Ramteke [29] observed that protease production by *Stenotrophomonas maltophilia* was not interrelated with growth and it reached a peak of 49 U/ml at 120 h of incubation. For *Bacillus subtilis* and *Bacillus* sp., the highest protease production was observed at 110 h and 30 h of incubation respectively [30, 34]. The protease activity of another strain of *Bacillus* sp. was correlated with cell growth which increased sharply at 16 h and reached a maximum after 60 h of incubation [12]. Maximum alkaline protease production by *Stenotrophomonas* sp. was reported at 32 h of incubation [28].

#### Incubation temperature

Temperature is a significant parameter that has to be controlled and varies greatly between cold-adapted microorganisms for maximum protease production and cell growth. Both free and immobilized cells by *Cryptococcus victoriae* showed maximum protease and biomass production at 15 °C [25]. A similar temperature of 15 °C was required for maximum protease production by *Bacillus subtilis* [30] and *Pedobacter cryoconitis* [61]. For *Chryseobacterium* sp., the optimal temperature for growth and protease production was 28 °C and 5 °C respectively [32]. The maximum temperature for growth and protease production by *Planococcus* sp. was 25 °C and 20 °C respectively [60]. A similar temperature of 20 °C was required for maximum protease production by *Bacillus* sp. [34].

#### Culture pH

It is well known that enzymatic processes and transportation of various components across the cell membrane are influenced by culture pH. Generally, most of the cold-adapted microorganisms are known to yield maximum enzyme in neutral to alkaline pH range. The maximum protease production for both free and immobilized cells by *Cryptococcus victoriae* was optimal at pH 8.0 [25]. The specific enzyme activity in *Chryseobacterium* sp. was found to be highest at pH 7.0 [32]. For *Bacillus* sp. and *Bacillus subtilis*, maximum protease was secreted at an alkaline pH of 9.0 and 10.0 respectively [30, 34]. The highest protease yield of 62.2 U/ml by *Stenotrophomonas maltophilia* was attained at pH 9.0 [29].

#### Effect of various metal ions

Cold-adapted protease production has also been affected by the presence of various metal ions in the media. Mageswari et al. [32] reported that a concentration of 0.02% ZnSO_4_ and CaCl_2_ in the medium resulted in increased protease production, whereas FeCl_3_ and KCl had no influence on protease production by *Chryseobacterium* sp. For *Stenotrophomonas* sp., Mg^2+^, Mn^2+^, and Ca^2+^ at a concentration of 5 mM displayed maximum protease production, but Zn^2+^, Cu^2+^, and Co^2+^ robustly repressed the protease production. Moreover, the combined supply of Mg^2+^, Mn^2+^, and Ca^2+^ showed enhanced protease production than adding them independently [28].

Furthermore, finding the relationship between various variables is necessary to develop an economical and optimal bioprocess for the industrial interest of cold-adapted proteases. The demand for cold-adapted enzymes and their related products is ever-increasing, and large-scale fermentation of cold-adapted microorganisms can be very useful for meeting the industrial requirements. Classical methods have either been time-consuming or have not been able to find accurate optimum conditions. The *Colwellia* sp.-derived cold-adapted protease showed a 3.0-fold enhancement in production via response surface methodology [59]. Recently, Białkowska et al. [31] reported an approximately 4.0-fold increase in protease production by combining various sources via the regression model. In another study, Han et al. [13] reported a 15-fold improvement in protease production by *Pseudoalteromonas arctica* in a mineral optimized medium using a statistical approach and fed-batch culture. These advanced approaches can help in calculating the most favorable production conditions in any given set up, which can be very beneficial for large-scale production of cold-adapted proteases in the future.

### Biochemical properties of cold-adapted microbial proteases

In the past couple of decades, cold-adapted proteases from cold-adapted microorganisms have been studied comprehensively so that they can be applied for particular applications based on their properties. For industrial purposes, cold-adapted proteases should possess strong activity and stability under reasonably extreme conditions. Cold-adapted proteases have shown optimal activity over a broad range of temperature and pH, mostly depending on the character of protease-producing cold-adapted microorganisms. In recent times, the cardinal temperature of cold-adapted proteases by psychrophiles and psychrotrophs has been reported between 10 and 60 °C. The optimum pH for their activity typically ranges in between alkaline pH of 7.0–10.0, with few reports on acidic and neutral pH for the highest activity. An aspartic protease from *Geomyces pannorum* and the cold-adapted protease from yeast *Sporobolomyces roseus* showed highest activity in pH 3.0 and 4.0 respectively [15, 32]. A variety of metal ions, detergents, and reagents have shown the varying effect on protease activity, some of them behaving as inhibitors while some act as enhancers depending on their interaction with particular protease. The metal ion Mn^2+^ inhibited the activity of metalloprotease by *Colwellia* sp. [62] but enhanced the activity of serine protease by *Acinetobacter* sp. [63]. Based on effects of commonly used inhibitors like PMSF, EDTA, EGTA, and Pepstatin A, proteases are classified into serine, metallo, aspartic, and other types accordingly. The various significant properties of cold-adapted proteases are summarized in Table 2.

**Table 2:** Biochemical properties of cold-adapted proteases (published from 2010 onwards)

Microbial source	Protease type	Strong inhibitors	Stimulators/enhancers	Optimum temp./pH	Reference
*Acinetobacter* sp.	Serine peptidase	PMSF, EDTA, EGTA, 2-ME, Pepstatin A, DTT, and Hg^2+^	Ca^2+^, Mn^2+^, Na^+^, Zn^2+^	40/9	[63]
*Bacillus* sp.	Metalloprotease	Cu^2+^, Zn^2+^, Hg^2+^, EDTA, and SDS	Mg^2+^and Ca^2+^	40/7.4	[12]
*Bacillus* sp.	Metalloprotease	EDTA, EGTA, Ca^2+^, Cu^2+^, Mg^2+^, K^+^, Zn^2+^	Mn^2+^	20/9	[34]
*Bacillus subtilis*	Serine protease	Mg^2+^, Pb^2+^, Mn^2+^, Al^3+^, and Fe^2+^	Cu^2+^ and Ca^2+^	15/10	[30]
*Chryseobacterium* sp.	Serine protease	Butanol, acetonitrile, isopropanol, ethyl acetate, tetrahydrofuran, Hg^2+^, Zn^2+^, and Cu^2+^	Na^+^, Ca^2+^, Ba^2+^, and Fe^2+^	10/7.0–8.0	[32]
*Geomyces pannorum*	Aspartic protease	Methanol, isopropanol, DMSO, Mg^2+^, Fe^2+^, Ca^2+^, and Zn^2+^	Co^2+^, Mn^2+^, Cu^2+^, and Ni^2+^	60/3.0	[15]
*Halobacillus* sp*.*	Thermolysin-like protease	EGTA, Cu^2+^, EDTA, 1,10-phenanthroline, and Ni^2+^	Mn^2+^, Ca^2+^, Mg^2+^, and Ba^2+^	30/8	[37]
*Lysobacter* sp.	Serine peptidase	PMSF, EDTA, and Zn^2+^	Ca^2+^, Mg^2+^, Ba^2+^, Na^+^, NH_4_^+^, and isopropyl alcohol	40/9.0	[18]
*Penicillium nalgiovense*	Serine protease	PMSF, SDS, Mn^2+^, and Zn^2+^	Ca^2+^ and Mg^2+^	35/8	[40]
*Planococcus* sp.	Serine protease	PMSF, DEPC, EDAC, urea, SDS, EDTA, Co^2+^, Zn^2+^, Fe^3+^, and Ni^2+^	Ca^2+^	35/10	[23]
*Planococcus* sp.	Serine protease	EDTA, PMSF, TNBS, EDAC, Cu^2+^, and Ni^2+^	Fe^3+^ and Ca^2+^	35/10	[60]
*Planomicrobium* sp.	Serine protease	PMSF and AEBSF	Ca^2+^ and Mn^2+^	35/9	[11]
*Pseudomonas aeruginosa*	Serine protease	PMSF and Ag^+^	Mg^2+^, K^+^, Ca^2+^, Ba^2+^, and Zn^2+^	25/10	[21]
*Pseudoalteromonas* sp.	Serine protease	PMSF, SDS, and H_2_O_2_,	Nm	25–35/8–9	[16]
*Pseudoalteromonas arctica*	Subtilisin-like protease	Linear alkylbenzene sulfonate (LAS) and SDS	Ca^2+^	30/9.0	[64]
*Pseudomonas lundensis*	Metalloprotease	EDTA, EGTA, Cu^2+^, Fe^3+^, Al^3+^, Fe^2+^, Mn^2+^, Al^3+^, and Co^2+^	Na^+^, K^+^, and Li^+^	30/10.4	[19]
*Serratia marcescens*	Metalloprotease	EDTA, MnCl_2_, CaCl_2_, CoSo_4_, HgCl_2_, and Na_2_	Nm	40/8	[27]
*Sporobolomyces roseus*	Aspartic protease	2-Mercaptoethanol, dithiothreitol, SDS, and Pepstatin A	Nm	50/4	[31]
*Stenotrophomonas* sp.	Alkaline protease	Zn^2+^, Cu^2+^, and Co^2+^	Mg^2+^, Mn^2+^, and Ca^2+^	15/10	[28]
*Stenotrophomonas maltophilia*	Alkaline protease	Co^2+^	Cu^2+^, Cr^2+^	20/10	[29]

*Nm* not mentioned

The stability of proteases under the wide range of temperature and pH is essential for their industrial applications, especially as detergent additives. The *Planococcus* sp.-derived cold-adapted protease was stable at 10 °C for 2 h and in a broad pH range of 5.0–12.0 for 30 min. However, there was a 93% activity loss at 35 °C after 2 h, but 80% of activity was retained within the pH range of 5.0–12.0 after 30 min [23]. For *Bacillus subtilis* protease, stability was observed in alkaline pH of 7.0–11.0 for 1 h and retained 63% of activity at 30 °C for 3 h at pH 10.0 [30]. Alkaline protease from *Stenotrophomonas* sp. was stable in the pH range of 6.8–12.0 and the temperature range of 15–30 °C for 1 h at pH 10.0, retaining 90% of activity in both conditions [28]. The cold-adapted proteases from diverse cold-adapted microorganisms vary in thermal and alkaline stability and have been reviewed from time to time [15, 21, 60, 63]. These broad-spectrum properties of cold-adapted proteases make them an interesting candidate for numerous applications under diverse conditions.

### Purification of cold-adapted microbial proteases

Cold-adapted protease producers are initially recognized based on different screening techniques followed by purification of their enzymes up to different levels. The molecular weight is usually determined via SDS-PAGE. Cold-adapted proteases vary extensively in their size range, the lowest being reported from *Bacillus amyloliquefaciens* protease, i.e., 23 kDa [65], and highest from *Curtobacterium luteum* protease, i.e., 115 kDa [66]. They are subjected to multistep techniques for attaining various levels of purification folds. Mostly, ammonium sulfate precipitation has been used for initial concentration, but in some cases, ultrafiltration and acetone precipitation have also been used. Within the past few years, novel techniques have been applied for increasing the yield percentage and purification fold of cold-adapted proteases (Table 3). The importance of purified cold-adapted proteases has been frequently endorsed in a variety of applications at the biotechnological scale.

**Table 3:** Molecular weight and purification of cold-adapted proteases (published from 2010 onwards)

Microbial source	Molecular weight	Chromatographic techniques	Purification fold/final yield	Reference
*Acinetobacter* sp.	35	DEAE cellulose and Sephacryl S-200	9.8/0.16	[63]
*Bacillus* sp.	62	Nm	3.82/76	[34]
*Bacillus subtilis*	38	DEAE cellulose	49.22/29.28	[30]
*Halobacillus* sp*.*	35	DEAEeSephadex, ion exchange, and Sephadex G-75 gel filtration	3077 ± 49/26	[37]
*Lysobacter* sp.	35	Nm	2.40/95.6	[18]
*Planococcus* sp.	35.6	His-Bind resin affinity chromatography	Nm	[23]
*Planococcus* sp.	43	DEAE-Sepharose	Nm	[60]
*Penicillium nalgiovense*	45.2	Nm	12.1/82.9	[40]
*Pseudomonas lundensis*	46	Gel filtration	14/20	[19]
*Pseudomonas aeruginosa*	32.8	DEAE-Sepharose and Sephacryl S-200 gel filtration	10/60	[21]
*Pseudoalteromonas* sp.	34.6	Affinity chromatography	Nm	[16]
*Serratia marcescens*	56	DEAE cellulose Fraction	9.9/51	[27]
*Sporobolomyces roseus*	31	HiTrap SPFF, Superose 12 and Mono S	103/25	[31]
*Stenotrophomonas* sp.	55	DEAE-Sepharose	18.45/47	[28]
*Stenotrophomonas maltophilia*	75	DEAE cellulose column	Nm	[29]

*Nm* not mentioned

### Substrate specificity of cold-adapted microbial proteases

One of the imperative features of cold-adapted proteases is their capability to distinguish between competing substrates, and the function of these cold-adapted enzymes is highly dependent on their substrate specificity. In general, cold-adapted proteases are known to be active against various native proteins and different types of natural and synthetic substrates, displaying wide substrate specificity. The extracellular cold-adapted alkaline peptidase produced by *Acinetobacter* sp. exhibited substrate specificity towards various protein substrates. The highest activity was found towards casein and BSA followed by azocasein and skim milk, whereas gelatin showed the lowest activity [63]. A thermolabile subtilisin-like protease (P6) from *Pseudoalteromonas* sp. hydrolyzed synthetic substrate—Succ-AAPF-pNa—and natural substrate—succinylated casein—but showed no activity on Succ-AAVA-pNa [16]. Another thermolysin-like protease (HSPA) secreted by *Halobacillus* sp. hydrolyzed various soluble and insoluble proteins, such as feather, elastin, collagen, hemoglobin, and BSA but proteins such as casein and gelatin were found to be suitable substrates. Among various synthetic substrates tested, HSPA exhibited higher hydrolytic activity on FA-Gly-Phe-NH_2_ and FA-Gly-Leu-NH_2_, but almost no activity was observed on FA-Ala-Arg-OH and FA-Glu-Glu-OH. Results signify that protease HSPA preferred Leu more than Phe at the P_1_′ position and demonstrated lower affinity towards alkaline and acid P_1_′ residues [37]. The hydrolytic activity of a cold-adapted serine protease produced by *Chryseobacterium* sp. was highest with casein followed by gelatin and BSA, whereas egg albumin was least hydrolyzed [32]. For cold-adapted serine peptidase by *Lysobacter* sp., the highest activity was observed on azocasein followed by gelatin and feather powder, whereas casein, BSA, and azokeratin showed lowest enzymatic activity [18]. An aspartic protease produced by psychrotrophic yeast *Sporobolomyces roseus* showed the highest activity against natural protein substrates such as urea-denatured and native hemoglobin, but lower activity was observed against synthetic substrates such as N-succinyl-Ala-Ala-Pro-Phe-p-nitroanilide and N-succinyl-Ala-Ala-Pro-Leu-p-nitroanilide [31]. The specific activity of another aspartic protease produced by psychrophilic fungus *Geomyces pannorum* was highest towards hemoglobin followed by κ-Casein and lowest towards cytochrome c [15]. The broad substrate specificity of cold-adapted proteases can be valuable for industrial applications, especially in bioremediation processes carried at low temperatures.

### Recent progress in gene cloning and protein engineering of cold-adapted microbial proteases

The extensive utilization of proteases in various industries has increased the requirement of novel cold-adapted proteases that possess high catalytic efficiency and thermostability together at low temperatures. Generally, higher catalytic efficiency at lower temperatures leads to weaker thermostability within most of the cold-adapted proteases, a common setback that deters their scope of utilization in industries. Keeping that in view, various new approaches like protease gene cloning, protein engineering, deletion mutagenesis, direct evolution, and site-directed mutagenesis have been successfully implemented to enhance the thermostability and catalytic proficiency of cold-adapted proteases. A subtilisin-like protease gene encoding a precursor protein was cloned and expressed in *E. coli*. The recombinant protein (P6) exhibited elevated catalytic efficiency than Carlsberg protease at a temperature range of 5–25 °C [16]. Similarly, the protease gene (*cpls8*) encoding an intracellular protease (CPLS8) from *Planococcus* sp. was cloned and expressed in *E. coli*. The recombinant CPLS8 illustrated remarkable alkali-stability at a pH range of pH 5.0–12.0 and higher catalytic efficiency at a temperature range of 5–35 °C which makes CPLS8 worthy as an industrial product [23]. The gene encoding cold-adapted serine protease (GpPro2) of *Glaciozyma antarctica* was cloned and expressed in *Pichia pastoris*. The recombinant GpPro2 displayed low thermostability and high catalytic activity at low temperatures, making GpPro2 an interesting candidate for biotechnological exploitation [17]. A cold-adapted peptidase gene (*a0301*) from *Lysobacter* sp. was heterologously expressed in *E. coli*, and recombinant A03Pep1 showed characteristics suitable for industrial applications [18]. Park et al. [64] cloned the *pro21717* gene encoding the psychrophilic serine protease (Pro21717) from *Pseudoalteromonas arctica*, and the recombinant Pro21717-CD exhibited higher activity at alkaline pH and low temperature. Moreover, Pro21717-CD showed stability against various chemicals and detergent surfactants, making it a valuable product for commercial detergent formulations. In a recent study, the gene *Alp* encoding serine alkaline protease of the psychrotrophic bacterium *Acinetobacter* sp. was cloned and expressed in *E. coli*. The recombinant protease (Alp) showed resistance to extreme alkaline conditions and low temperatures suggesting its potential in the detergent industry [67]. A novel aspartic protease gene *P10* from *Geomyces pannorum* was cloned and heterologously expressed in *Aspergillus oryzae*. The recombinant *P10* showed improved stability and potential application in cheese-making [15]. Successful cloning of the cold-adapted alkaline protease gene from *Bacillus subtilis* [30], protease gene *hspa* from *Halobacillus* sp. [37], and protease gene *cpls41* from *Planococcus* sp. [60] has also been reported.

Earlier, Yan et al. [68] cloned and expressed the *mcp-03* gene encoding cold-adapted halophilic protease (MCP-03) of the psychrotolerant *Pseudoalteromonas* sp., and the recombinant MCP-03 was more thermolabile and active than Carlsberg subtilisin at low temperatures. The deletion mutagenesis illustrated that the C-terminal PPC domains were obligatory for the higher thermostability of MCP-03 but affected the catalytic efficiency and caused restraint in the activity. However, recently, Zhao and Feng [69] engineered several variants of mesophilic alkaline serine protease from *Bacillus pumilus* by a combination of direct evolution and site-directed mutagenesis. The P9S/K27Q and P9S/T162I variants showed a 2.6-fold improvement in catalytic efficiency (kcat/km) and 5-fold enhancement in specific activity respectively than wild-type enzyme at 15 °C, without showing any negative effect on thermostability. The results suggested that by advanced engineering techniques, it is possible to improve both catalytic efficiency and thermostability together at the same time without causing any restraint on each other’s activity.

### Structural analysis and molecular modeling of cold-adapted microbial proteases

The principal objective regarding the structural study of cold-adapted proteases has been to develop a better understanding of their adaptation to cold temperature environments. In the past, various solved crystal structures of cold-adapted proteases have been reported that explain the structure-environment adjustment of proteins and provide valuable insights that are important for exploiting their industrial and therapeutic potential. Historically and in the present time, crystallography and homology modeling have been extensively used to resolve the structures of cold-adapted proteases. Structural comparison of cold-adapted proteases with their mesophilic and thermophilic counterparts has provided constructive facts about the molecular basis of low-temperature adaptation [9]. Previous and recent reports conclude that cold-adapted proteases demonstrate more flexibility, have lengthier loops, have fewer salt bridges, are negatively charged amino acids, and are hydrophilic. They contain a higher number of glycine residues, whereas arginine and proline are present in lower concentrations. The surface of three-dimensional structures probably has a higher percentage of hydrophobic side-chains and contains more negatively charged residues than positively charged residues [4, 67, 70]. As the study on structural analysis of cold-adapted proteases progresses and several crystal structures are solved, the knowledge related to their low-temperature adaption keeps on advancing. A 3D model of subtilisin-like cold-adapted protease (P6) from *Pseudoalteromonas* sp. was built and compared with the mesophilic variant (P23314) from *Xanthomonas* via homology modeling. By the combination of local packing analysis and site-directed mutagenesis, it was determined that the Ala residue might be responsible for cold-adaptation of P6. Moreover, the Ala residue via laboratory evolution was found to be accountable for cold adaptation of the mesophilic P23314 also. Therefore, evaluating the position of this Ala residue might provide valuable information about substrate specificity and temperature adaptation of subtilisin-like proteases [16]. In another study, a homologous model of *Planococcus* sp.-derived cold-adapted protease (CPLS8) was built using Swiss-model by comparing the crystal structure of subtilisin from *Bacillus clausii*. Furthermore, the secondary and tertiary structures of CPLS8 were analyzed, predicted, and compared with three different structures of meso-subtilisin protease (MSP), thermo-serine protease (TSP), and a thermostable serine protease (ETSP). The intramolecular interactions of CPLS8 were found to be weak, and loosely packing was observed as compared to meso- and thermo-counterparts. In comparison with TSP and ETSP, the CPLS8 had a higher number of amino acid residues and a reduced number of hydrogen bonds. These differences possibly explain the low-temperature adaptation of CPLS8 [23]. Pereira et al. [18] determined the crystal structure of a cold-adapted serine peptidase (A03Pep1) from *Lysobacter* sp. The comparative studies illustrated that A03Pep1 had less deep and wider binding pocket than mesophilic peptidase AprV2; this difference might be due to higher activity of A03Pep1 at low temperatures. Recently, a crystal structure of catalytic domain from cold-adapted protease (Pro21717) of psychrophilic *Pseudoalteromonas arctica* was determined at a resolution of 1.4 Å. The Pro21717-CD structural analysis concluded that a co-purified peptide at the substrate-binding site showed unanticipated electron density which gave an idea about the substrate recognition and binding mode of cold-adapted Pro21717. Several other factors like rich active-site loop content, broad substrate pocket size, and structural flexibility assisted further in the better understanding of low-temperature adaptation and industrial potential of Pro21717 [64]. These reports demonstrate that structural analysis and molecular modeling of cold-adapted proteases provide a better understanding of their low-temperature adaptation, which can be exploited for improving their quality and scope in various industries. Moreover, comparing the structures of cold-adapted proteases with their hyperthermophilic counterparts can also be considered in the future for more valuable insights and deeper understanding related to low-temperature adaptation.

### Prospective applications of cold-adapted microbial proteases

Modern-day biotech industries need enzymes that are eco-friendly and economically beneficial, and cold-adapted proteases likely have that industrial and biotechnological potential in them. They have gained popularity over the last few decades due to the recognition of their catalytic capability at low temperatures and low thermostability at high temperatures. With advanced research, the future of cold-adapted proteases promises a higher industrial market compared to mesophilic and thermostable proteases. The recent applications of cold-adapted proteases reported for various industrial sectors are described below and are also presented in tabular form (Table 4).

**Table 4:** Prospective applications of cold-adapted proteases from psychrophiles and psychrotrophs

Microbial source/protease	Potential application	Reference
*Acinetobacter* sp. (serine protease)	Suitable for detergent formulations	[63]
*Arsukibacterium ikkense* (cold-active protease)	Applicable in dairy products and other functional foods	[22]
*Bacillus* sp. (metalloprotease)	Detergent additive for cold-washing	[34]
*Bacillus* sp. (metalloprotease)	Environmentally friendly feed additive to improve the production performance of farm animals	[12]
*Bacillus subtilis* (alkaline protease)	Biodegradation of protein rich wastes	[71]
*Bacillus subtilis* (serine protease)	Detergent additive for cold washing	[30]
*Chryseobacterium* sp. (serine protease)	Applicable in meat and other food processing units	[32]
*Enterococcus faecalis* (metalloprotease)	Improves the stability and solubility of health foods	[72]
*Flavobacterium limicola* (cold-active protease)	Primary mineralization of organic polymers in freshwater sediments	[73]
*Pedobacter cryoconitis* (metalloprotease)	Bioremediation of wastewater in cold conditions	[61]
*Penicillin nalgiovense* (alkaline protease)	Suitable for meat ripening purposes	[40]
*Planococcus* sp. (serine protease)	Detergent additive for cold washing	[60]
*Pseudomonas aeruginosa* (alkaline protease)	Cold washing detergent enzyme	[21]
*Pseudoalteromonas* sp. (serine protease)	Improves the taste of refrigerated meat	[74]
*Pseudoalteromonas* sp. (serine protease)	Applicable in low-temperature food processing	[59]
*Pseudoalteromonas arctica* (subtilisin-like protease)	Suitable for cold-active laundry or dishwashing purposes	[64]
*Serratia marcescens* (metalloprotease)	Detergent additive for cleaning purposes	[27]
*Stenotrophomonas* sp. (alkaline protease)	Suitable for detergent and textile industry	[28]

#### Food industry

Cold-adapted proteases have shown promising prospect in the food industry due to the fact that they are thermally unstable and can be selectively and rapidly inactivated when required. Moreover, these cold-adapted enzymes are beneficial due to their optimal enzymatic activity at low temperatures which eliminates the risk of microbial contamination [75, 76]. He et al. [74] reported that cold-adapted protease of *Pseudoalteromonas* sp. improved the taste of frozen meat better than mesophilic protease by releasing extra taste amino acids and essential amino acids. Another psychrophilic *Pseudoalteromonas* sp.-derived cold-adapted protease released more free amino acids from milk protein in contrast to mesophilic papain at 4 °C, suggesting the extensive substrate specificity and prospective function in low-temperature food processing [59]. The cold-adapted metalloprotease from *Enterococcus faecalis* has been proven safe for oral administration with no side effects at all. This enzyme can be functionally used in the food industry by direct means and can improve the stability and solubility of health foods [72]. An alkaline peptidase from *Penicillium chrysogenum* showed the potential to be more valuable for cheese manufacturing compared to the commonly used microbial mesophilic and thermostable proteases [77]. Similarly, an aspartic protease from *Geomyces pannorum* showed features that are suitable for cheese-making [15]. The proteolytic enzymes secreted by *Arsukibacterium ikkense* produced bioactive peptides by degrading casein extensively, and these enzymes were suggested to be appropriate for dairy products and other functional foods [22]. The cold-adapted serine protease from *Chryseobacterium* sp. showed diverse properties such as low-temperature activity and salt tolerance and was potentially applicable in meat and other food processing industries [32].

#### Detergent industry

Proteases with high levels of activity at low temperatures came into existence after certain limitations were found in thermostable proteases. These low-temperature proteases were introduced at the commercial level in the year 1985 as third-generation proteases [78]. The main purpose behind developing such low-temperature proteases was energy and time preservation. Besides alkali stability, a good detergent protease is expected to be stable in the presence of commercial detergents, oxidants, and surfactants. Microbial cold-adapted proteases along with detergents have proven to be more effective during cold washing as compared to enzyme-free detergents. At lower washing temperatures, numerous cold-adapted proteases have shown outstanding activity and stability in broad alkaline pH as well as compatibility with a variety of commercial detergents [30, 34, 60, 63, 64]. Also, cold-adapted alkaline proteases have shown excellent stability in commercially available surfactants and bleaches [21]. Furthermore, cold-adapted proteases along with commercial detergents have removed the proteinous matter from clothes stained with chocolate, tea, blood, egg yolk, grass, etc. at low temperatures much more efficiently as compared to enzyme-free detergents. These properties make them suitable as appropriate detergent additives for laundry industries and dishwashing purposes.

In recent times, Palo Alto (CA, USA) released two cold-adapted detergent proteases (Purafect® and Properase®) in the market which are active at low temperatures. Another cold-adapted detergent additive protease with market name Excellase® developed by Genencor has been launched in liquid form for dishwashing purposes [79].

#### Textile industry

Cold-adapted proteases might find applications in the textile industry because their actions on fabrics can reduce the harmful effects of chemicals. They can enhance the life of woolen and silk fabrics by retaining the quality of cloth after washing at lower temperatures. Reports suggest that cold-adapted protease treatment can improve the surface appearance as well as reduce the bristles of woolen fabrics and preserve the finishing of silk cloths [27, 28].

#### Feed additives

Proteases due to their extensive substrate specificity as well as reasonably advantageous activity levels at a physiologically applicable temperature and pH can be used as an eco-friendly feed additive for improving the manufacturing performance of animal farms. Cold-adapted proteases which possess keratinolytic activity can facilitate and endorse biotechnological processing of biomaterials consisting of keratinous waste from leather and poultry industries [12].

#### Polymer degrading

Various species of genus *Flavobacterium* regulate their fatty acid composition during cold conditions which assists them in maintaining their membrane fluidity. This process helps them in degrading different organic polymers that facilitate the production of a considerable amount of extracellular protease in cold temperatures, thus playing a vital part in the prime mineralization of composite organic materials present in freshwater sediments throughout cold seasons [73].

#### Bioremediation

Proteases withstanding low temperatures may find applications in environmental biodegradation of protein-rich wastes and wastewater treatment in cold conditions [71]. Anaerobic psychrophiles from Antarctic surroundings possess an ability to thrive and produce proteases on a broad range of substrates which indicates their potential of being used for the breakdown of protein-rich substrates like night soil [80]. In another example, a psychrophilic *Pedobacter cryoconitis* utilized a large number of organic compounds such as oil hydrocarbons, carbohydrates, and proteins and was recommended to be suitable for the treatment of impure wastewater in cold conditions [61].

## Conclusion

Cold-adapted microbial proteases are mainly characterized by low thermostability at elevated temperatures and high catalytic effectiveness at cold temperatures at which meso-variants are not active. Regardless of the widespread investigation, the knowledge about these unique enzymes is still limited. The current biotechnological era demands more novel cold-adapted proteases from psychrophiles and psychrotrophs with exciting features for industrial and research purposes. There is a need for exploring new ways for economical and large-scale production of cold-adapted proteases. The positive effects of gene cloning and protein engineering have greatly influenced the quality and production of recombinant enzymes. Further, extensive efforts are required for identifying unique and novel cold-adapted protease genes that can be tailored to attain desired results. The quantitative economic overproduction of cold-adapted proteases can be achieved by strain improvement and optimization of various production parameters along with the identification of cheaper nutrient sources. Furthermore, solving more crystal structures can provide in-depth knowledge and a better understanding about the structure-function relationship of cold-adapted proteases. Mostly, mesophilic proteases have been used for comparative structural studies. In the future, thermophilic, hyper-thermophilic, and other distinct counterparts can also be considered to explain the changes that may be associated with cold-adaptation of psychrophily and psychrotrophy. To conclude, it is likely that the present review will help in giving better insights about protease biotechnology and help in attaining more robust cold-adapted proteases in the future.

## Data Availability

Data sharing is not applicable to this article as no datasets were generated or analyzed during the current study.
